# The Biomechanics of Pregnancy: A Systematic Review

**DOI:** 10.3390/jfmk4040072

**Published:** 2019-12-02

**Authors:** Rebecca Conder, Reza Zamani, Mohammad Akrami

**Affiliations:** 1 Medical School, University of Exeter, Exeter EX1 2LU, UK; r.conder2@gmail.com (R.C.); r.zamani@exeter.ac.uk (R.Z.); 2 Department of Engineering, College of Engineering, Mathematics, and Physical Sciences University of Exeter, Exeter EX4 4QF, UK

**Keywords:** pregnancy, biomechanics, gait and posture, review

## Abstract

During pregnancy, a number of biomechanical and hormonal changes occur that can alter spinal curvature, balance, and gait patterns by affecting key areas of the human body. This can greatly impact quality of life (QOL) by increasing back pain and the risk of falls. These effects are likely to be the ultimate result of a number of hormonal and biomechanical changes that occur during pregnancy. Research Question and Methodology: Using the Preferred Reporting Items for Systematic reviews and Meta-Analyses (PRISMA) guidelines, this systematic review sets out to analyse all available literature relating to the biomechanics factors caused by pregnancy and assess how this might reduce QOL. Fifty papers were deemed eligible for inclusion in this review based on the PUBMED and SCOPUS databases. Results: Angles of lordosis and kyphosis of the spine are significantly increased by pregnancy, but not consistently across all studies. Back pain is significantly increased in pregnant women, although this is not significantly correlated with spinal changes. Increased movements of centre of pressure (COP) and increased stability indexes indicate postural control is reduced in pregnancy. Trunk range of motion, hip flexion, and extension are reduced, as well as decreased stride length, decreased gait velocity, and increased step width; again, not consistently. It is likely that each woman adopts unique techniques to minimise the effects, for example increasing step width to improve balance. Further research should focus on how altered limb kinematics during gait might affect QOL by influencing the human body, as well as assessing parameters in all planes to develop a wider understanding of pregnant biomechanical alterations.

## 1. Introduction

According to a study carried out by Dunning et al. [1], 27% of pregnant women experienced a fall during their pregnancy. This highlights a risk to the safety and wellbeing of both mother and child. Further to this, it is estimated that around 56% of pregnant women experience lower back pain (LBP) at some point during their pregnancy [2]. These effects are likely to be the ultimate result of a number of hormonal and biomechanical changes that occur during pregnancy. For example, increased levels of relaxin are thought to be responsible for increased ligament laxity and thus changes in the musculature of the body, particularly in the lower trunk [3]. Changes to the spinal curvature in terms of both lordosis (inward curvature) and kyphosis (outward curvature) are also apparent [4]. Nevertheless, the effects of these differences in pregnant morphology are associated with alterations in sitting and standing posture [5]. They influence the balance (both statically and dynamically) [6], as well as changes in the kinematics of the limbs during gait [7] by virtue of ergonomic changes due to the pregnancy [8]. The kinematics widely discussed in the available literature include the altered hip, knee, and ankle movements in all planes, as well as alterations in the kinematics of the trunk relating to the relationship between the torso and the pelvis. In terms of posture, studies have shown significantly increased lordotic angles when comparing pregnant to non-pregnant women [4]. In terms of balance, changes to the center of pressure (COP) are used as an indicator of stability. Evidence shows that in many cases, COP in pregnant women shows significantly increased path length, which indicates reduced stability [6]. Gait is analysed in terms of spatiotemporal parameters, and many studies have reported an increase in step width and reduced gait velocity [9]. Changes to kinematics of the joints during gait are also reported, in which case many pregnant women display reduced flexion and extension in the hip, amongst other effects [7]. 

As mentioned, these changes can impact quality of life (QOL) in a number of ways, including increased reporting of pain [10,11], aversion to unsuitable ergonomic conditions [12], and increased risk of falls [13]. Risk of falling is particularly increased in occupations in loud environments [1]. The highest fall rates for pregnant women were observed in nurses, social workers, and waitresses [14]. Sleep is also shown to be affected in pregnant women as levels of insomnia and specific awakenings have been shown to increase with a developing pregnancy, which again has been reportedly accounted for by the hormonal and biomechanical effects of pregnancy [15]. These impacts are argued to cause reduced QOL for pregnant women.

Current therapeutics include the use of pelvic belts to combat pelvic girdle pain during pregnancy [16]. The literature also suggests methods of alleviating the risk, including wearing appropriate shoes with inserts for pain relief [17], exercises for back strengthening [18], and advice for employers to ensure working environments are safe for pregnant employees [1]. Despite these recommendations, it is important to further explore the effects of pregnancy in order to identify the challenges to tackle when creating new and improving already available therapeutics for pregnant women by means of ergonomic designs of the workplace suitable for pregnant women [19]. Compiling all the relevant available literature should give future researchers an idea of the risks in pregnancy that require further innovation to improve QOL and reduce potential pain or risks to the mother. 

This systematic review will explore the effects of pregnancy on the biomechanics and anthropometrics of the body and how this results in altered posture, stability, and gait patterns that influence the body. It can change the gait patterns, torso, and joint kinematics, which potentially affects the anatomical shape of the body. Furthermore, the papers will reveal changes in motions such as rising from a chair and forward flexion, as well as changes to pelvic-thoracic rotations and trunk control during gait. The results will be discussed in terms of the relationship with QOL for pregnant women. 

## 2. Materials and Methods

As a template for the methodology of this review, the Preferred Reporting Items for Systematic reviews and Meta-Analyses (PRISMA) guidelines were used [20]. 

### 2.1. Eligibility Criteria

Papers were included if they met the dual criteria of being written in English and being primary research articles in peer-reviewed journals. Exclusion criteria for this review are as follows: review articles, papers in which the participants have comorbidities, papers with a journal impact factor of Q3/Q4.

### 2.2. Selection Methods

Databases searched were PUBMED and SCOPUS. The last dated literature search was carried out on 14th January 2019. Eight hundred and thirty-three papers were revealed using keywords in a combination of four boolean algorithms for both databases. Note that the search engines were set to keywords being present in the title of the papers and no date restrictions were applied. To the best of the authors’ knowledge, there are no papers investigating the physiological changes of the body due to pregnancy using experimental techniques. 

The algorithms and the results they yielded are presented below.

1 (Pregnancy OR Pregnant OR Pregnancies) AND (Biomechanics OR Biomechanical OR Biomechanically)

Results from Pubmed: 225

Results from Scopus: 54

2 (Pregnancy OR Pregnant OR Pregnancies) AND (Posture OR Postural)

Results from Pubmed: 143

Results from Scopus: 185

3 (Pregnancy OR Pregnant OR Pregnancies) AND (Gait)

Results from Pubmed: 33

Results from Scopus: 49

4 (Pregnancy OR Pregnant OR Pregnancies) AND (Stability)

Results from Pubmed: 66

Results from Scopus: 78

**Figure 1:**
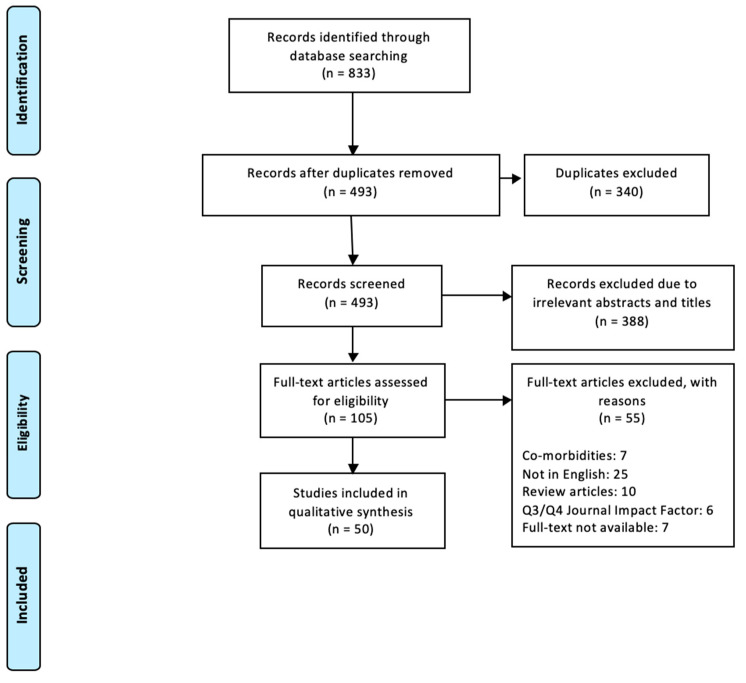
The flowchart showing the process of recording, screening and reviewing the articles using Preferred Reporting Items for Systematic reviews and Meta-Analyses (PRISMA).

Duplications were identified within the papers found, which included both duplicates between the databases as well as internal duplicates of the same research being published in more than one format. Removal of duplicates left 493 papers to be screened. Screening involved checking abstracts and titles for relevance to the review topic. Screening removed 388 papers deemed as irrelevant to this research, and the reasons for these removals are identified in Table 1. This left a total of 105 papers to be assessed against inclusion and exclusion criteria. After inclusion and exclusion criteria had been applied, 50 papers were deemed eligible for inclusion. The PRISMA flow chart outlines this process for finalising papers (See Table 2) as well as reasons for the final inclusions and exclusions (see Figure 1). 

## 3. Results and Discussion

### 3.1. Posture

#### 3.1.1. Spinal Curvature

Ordinarily, the human spine shows both inward and outward curvature. Lordosis (inwards curvature) is observed in the lumbar and cervical regions of the spine. Kyphosis (outward curvature) is observed in the thoracic and sacral regions of the spine. Studies have shown that pregnancy significantly increases the lordotic angle of the spine [21], as well as the kyphotic angle in the developing pregnancy between trimesters two and three [4,22]. Other studies report findings of increased lordosis in the third trimester of pregnancy (preceded by a small reduction between trimesters one and two), but no changes in kyphotic angles throughout pregnancy or compared to non-pregnant control participants were reported [23,24]. Theoretically, this would make sense in terms of pregnancy since the growing abdomen is located closest to the lumbar spine, and thus lordosis should be most affected. However, Betsch et al. [25] found that there was no change in lordotic angle during pregnancy but an increase in kyphosis was observed both in the developing pregnancy and when comparing pregnancy results to postpartum results. Another study observed no changes in either kyphosis or lordosis angles throughout pregnancy [26]. Across these studies, there is an obvious conflict between findings, while some participants show deviations from the overall findings. Therefore, it is likely that spinal curvature of the participants varies depending on the individual. This difference could be due to factors including body weight and tendency to exercise. A reduction in kyphosis of the spine has been significantly correlated with greater body weight in pregnant women [27]. Meanwhile, lordosis is shown to exhibit a slight reduction after trimester two in pregnant women enrolled in at least three exercise classes per week. However, other correlations between posture and exercise are shown to be minimal [23]. The degree of pelvic tilt has also been shown to change in pregnant women, while the results vary among studies. Pelvic tilt has been shown to be significantly more anterior in pregnant women in their third trimester, in comparison to non-pregnant control women [28], which can cause instability. Similar results between the first and second trimester of pregnancy were observed by other researchers [22], showing a slight reduction in pelvic tilt, followed by a slight increase between the second and third trimester. However, these results were found to be statistically insignificant. Regarding the postpartum, the pelvic tilt is less anteriorly tilted compared with the non-pregnant control women in a seated position. Other studies report no significant findings in terms of changes to pelvic inclination in pregnant women [25,29]. The available literature discusses the idea that the tilt of the pelvis may be associated with alterations in the spine. Weakened abdominal muscles during pregnancy due to increased levels of relaxin and progesterone (which relax the muscles), or as a result of the overstretching of the muscles due to increased abdominal size, are thought to be responsible for an increase in anterior pelvic tilt [30]. The pelvis is connected to the lumbar spine via a group of muscles known as “hip flexors”; an anteriorly-tilted pelvis shortens the hip flexors and increases lordosis of the spine [31,32,33]. This supports the results that report increased lordosis, particularly in the third trimester, since this is the point of greatest stretch of the abdominal muscles [4]. 

Several studies also assess the association of spinal curvature with pain levels in pregnancy [21,22,24,25,26,34], reporting that levels of low back pain increase with the developing pregnancy [4]. It is concluded that 95% of participants experienced LBP, which correlates with increased lordosis [24]. Another study analysed that 83% of participants experienced LBP during their pregnancy [21]. Although this coincided with significantly increased lordosis in the study, no significant link was made. Studies that assessed pain were shown to exclude smokers from their data, since smoking is associated with higher levels of pain [23,24].

Conflicting results from these studies could result from the variation in methodologies applied. The aforementioned studies assessed spinal curvature by use of digital photography [35]. Others use computerised methods [21], while some others made use of surface topography to produce 3D computational models of the spine [25]. All studies focus on analysing these based on the sagittal plane. It might be worth exploring further whether there are changes in the frontal plane in a conjoint perspective. If so, these may show more consistent associations between individuals, as well as a better explanation for the reports of back pain. 

#### 3.1.2. Trunk Range of Motion

Studies suggest that the inclination of the trunk during pregnancy increases with the developing pregnancy [29]. Nicholls et al. [36] reported that this was not consistent in two participants, as they displayed no trunk inclination changes. This is concordant with data from other studies, which has shown no changes to trunk inclination with pregnancy [27]. This suggests that despite general trends, posture in this aspect again depends on the participants’ characteristics. In a study assessing standing working posture, results showed that pregnant women adopted a trunk lean that was further backwards, and in doing so the hips also moved further backwards [37]. Reduced inclination of the trunk has been shown to be associated with increased levels of pain [25]. Therefore, it is possible that women increase their trunk lean backwards in an effort to reduce discomfort. Meanwhile, a study assessing sitting posture established that the angle of the trunk was on average larger in pregnant women as the upper trunk was more curved, thus supporting reports of increased degrees of kyphosis in pregnancy [38].

In a seated position, when tasked with reaching down forwards, lumbar flexion is significantly reduced in pregnant women compared to non-pregnant women, however no effect of pregnancy is observed here in terms of lateral bending [39,40]. Furthermore, the strength of the flexion is seen to be greater postpartum, while the lumbar extension strength is greatest during the second-trimester measurements [27]. In terms of standing flexion, results show that when standing at a table, forward flexion of the trunk increases by around 11 degrees on average over the duration of pregnancy, which concords with an increased elevation of the upper arm [41]. In this case, the variation between individuals increases with pregnancy, which again highlights the likelihood of changes being specific to individuals [41]. The moment of inertia when looking at the trunk and its movement in the y-axis in pregnant women is shown to be significantly larger. This translates as the increased tendency of the trunk to resist movement since the trunk’s displacement in the y-axis is significantly lower during pregnancy compared to control women [42].

### 3.2. Stability

#### 3.2.1. Static Stability

Studies investigating static stability carry out measurements using force plates that allow perturbations during measurements for the patterns of the centre of pressure (COP) and centre of mass (COM). Pregnancy reduces stability significantly in the third trimester of pregnancy, which is revealed by increased path lengths and area of COP [6,43,44]. In some cases, these increases of COP and thus instability were observed in second-trimester women, but in all cases no significant changes were found in the first trimester compared to non-pregnant women [6,13,43]. Weight distribution index (WDI) scores significantly increase in the third trimester of pregnancy, which, contrasting with other evidence, suggests that the balance improves as the pregnancy develops. However, WDI scores in pregnant women were still lower than control women, showing that pregnancy does hinder stability [4]. COM has been shown to move more anteriorly in pregnant women, but no changes have been seen here laterally [42]. Visual cues have been extensively studied in static stability. When women are asked to keep their eyes open, stability has been shown to improve [43] and in conditions that require women to keep their eyes closed, path length of the COP is increased by pregnancy [13,45,46]. It is reported that the condition of closing eyes affected both pregnant and non-pregnant women in the same way, and it is concluded that the destabilisation is due to poor somatosensory processing rather than anatomical changes of pregnancy [45]. This highlights the importance of visual cues for the maintenance of balance. Interestingly, in instances where the eyes are closed, sufficient balance has instead been maintained by spreading the feet apart [43,46]. The idea here is that increasing the width of the stance increases the base of support and therefore is an attempt to improve stability. Increased sway was only significant in the anteroposterior (AP) direction [47]. This study suggests that the lack of findings in the mediolateral (ML) direction is due to the increased stance width, which improves lateral balance.

Higher levels of anxiety have been positively correlated with increased levels of sway [47]. However, no significant differences have been reported when comparing ‘high anxiety’ pregnant women to ‘low anxiety’ pregnant women. In women who experience lower back pain (LBP) during pregnancy, higher stability indices are observed than for pregnant women who do not experience LBP. This suggests LBP further reduces balance during pregnancy, supported by results that show higher fall indices in LBP patients [48]. 

Across these studies, stability is commonly associated with an increased fall risk. It is reported that pregnant women are more prone to experiencing a fall than non-pregnant women, with studies showing around 25% of pregnant women fall at some point during their pregnancy [1,6]. Inanir et al. [13] directly correlated an increase in ‘fall risk test score,’ with significantly increased measures of antero-posterior stability index (APSI), overall stability index (OA), and mediolateral stability index (MLSI) in third trimester women. This indicates poorer postural control as a result of pregnancy. Takeda et al. [49] recorded that in women who fell during pregnancy, the back rectangular area of movement of the COP was greater compared to women who had not experienced a fall. A fear of falling may increase levels of caution in pregnant women, which may influence gait patterns. 

In women who fall during pregnancy, ankle stiffness is seen to be reduced compared to pregnant women who have not experienced a fall [50]. Ersal et al. [50] exposed women to anteroposterior perturbations using the force plate and noted that a large shift in COP is required as a method of correction to oppose the force. It was observed here that pregnant fallers showed reduced peak COP values compared to non-fallers and non-pregnant women. This suggests that greater ankle stiffness in pregnant women is beneficial in creating a force to counteract the perturbations and improve balance. Since ankle stiffness varies between individuals, calculating this parameter in the individual may be useful for pregnant women to evaluate their risk of falling.

#### 3.2.2. Dynamic Stability

To obtain data for dynamic stability, studies required pregnant women to perform gait cycles upon walkways fitted with force plates to obtain measurements for the COP. Results generally showed that with developing pregnancy, the mediolateral (ML) COP shift increases [7,29]. However, one study reported that both anteroposterior (AP) and ML shift of COP are reduced in pregnant women in comparison to non-pregnant women [51]. They are even reduced in women who experience pelvic girdle pain (PGP). Findings also report that as gait speed increases, the velocity of COP excursion increases [9], while others report that the area of COP excursion is reduced with increasing gait speed [51]. Furthermore, evidence suggests that an increased step length significantly correlates with a lower stability index in pregnant women [29]. COP deviation varies between different stages of the gait cycle, as COP movement in the forefoot contact phase is reduced by pregnancy while COP deviation in the flat foot phase is increased by pregnancy [7]. No significant changes to COP with the developing pregnancy were found [9]. Certainly, the differences in centre of pressure for the pregnant and normal subjects can be further analysed using computational models [52] under dynamic loading conditions. 

Research has also been conducted into stability during the act of rising from a chair and results have shown that the vertical velocity of the COM movement peaked significantly earlier but was lower in pregnant women compared to non-pregnant women. This indicates pregnant women begin to stand earlier but more slowly when asked. However, there is no significant effect of the developing pregnancy on COM changes when rising from a chair [53].

These experimental studies were applied using different types of technique. Among the 5 studies found that assessed dynamic stability, methodologies varied. Two different walkways were used in these studies: the VICON-3D motion system and the GAITRite walkway [7,9,51,53] with project-specific setups, while another study utilised a different camera motion capture system, Qualisys [29]. Besides the evident differences among the subjects, different commercial systems for analysing human motion [54] might be a potential explanation for variation between the results. 

### 3.3. Gait 

#### 3.3.1. Spatial and Temporal Parameters

A single gait cycle can be separated into the stance phase and the swing phase. The stance phase begins with the first moment of contact of the foot with the floor and continues while the foot remains in contact with the floor. The swing phase begins the moment the foot leaves the floor: this moment is defined as ‘toe-off.’ Any point when both limbs are touching the floor is determined as ‘double support,’ and any time when only one limb has contact with the floor is defined as ‘single support’ [55]. Spatially, in pregnancy a decreased stride length is observed [56,57,58]. Alongside this, pregnant women display an increased step width, which is shown to be at its greatest during the third trimester [23,44,57,58,59,60]. This step width reduces again postpartum [57]. The literature extensively discusses the idea that increased step width is a method used by pregnant women to increase their base of support and therefore increase their stability during gait [57], which is also applicable to static conditions. One study also showed that the foot orientates itself more towards the outside during pregnancy [58]. Despite these significant findings, similar studies have reported no effect of pregnancy on stride length or stride width [29,61,62]. Temporally, gait velocity is reduced by pregnancy [4,56,59]. However, researchers found that at slower speeds, pregnancy had no effect on gait velocity, and that pregnancy reduced velocity only at higher speeds [58]. During pregnancy, single limb support time was shown to decrease while double support time increased [56,58,59,60,63]. Pregnant women also display shorter swing phases and longer stance phases in comparison to non-pregnant women [56,58,59]. A significant correlation is seen between a decreasing stride length and a decreased gait velocity amongst pregnant women [56]. Pregnancy-related pelvic girdle pain (PPP) is shown to further reduce the velocity of gait when compared to healthy pregnant women [64].

Alongside testing gait changes at high and low speeds, women are asked to walk at a speed most comfortable to them [57,65]. In these studies, lower velocities are observed in pregnant women; it is suggested that this could be the result of a fear of falling. It is likely that pregnant women may opt for a lower comfortable speed so as to reduce the risk of falling and injuring themselves. 

#### 3.3.2. Joint Kinematics

Studies show that there are significant reductions in the peak hip flexion and peak hip extension in the sagittal plane during the second and third trimester of pregnancy compared to non-pregnant women [7,63]. In the frontal plane, there is conflicting evidence whereby some studies report higher hip adduction in pregnant women during gait, whereas others report larger peak hip adduction angles in postpartum women [7,59]. This may be due to comparing pregnant women to different sorts of controls, where some studies use non-pregnant women and others use postpartum measurements of the same women. Decreased thigh abduction is observed in the developing pregnancy and compared to non-pregnant women [63]. In the transverse plane, peak external rotations (lateral and medial) of the hip are shown to be significantly higher in pregnant women, and these are at their highest in the third trimester. In an interesting study, Branco et al. reported that in terms of hip joint power, there are significant predictors in pregnant women [66]. It was observed that thigh fat area is a significant predictor of hip joint power during trimester two, while body weight is a significant predictor of hip joint power during pregnancy. 

The knee joint shows increased maximum flexion sagittally in the developing pregnancy, whilst displaying significant reductions in maximum extension of the knee when compared to non-pregnant women [59,63].

Regarding the ankle, increased inversion and eversion are observed in both the developing pregnancy and when compared to controls in the frontal plane [59,63]. This coincides with increased rotation of the foot during pregnancy, tending towards pronation. A significantly reduced plantar flexion is also observed during pregnancy [63]. However, there are also studies that have found no significant changes to the ranges of motion in the ankle, knee, and hip, including no changes to ankle inversion/eversion and knee flexion/extension [60].

It is likely that these kinematic effects are connected in some way, especially since it is known that an increased pelvic tilt can reduce the flexion moment in the hip [67]. The literature makes little reference to changes in the adduction of the knee joint. There is evidence suggesting that increased inversion and eversion in the ankle (observed in pregnancy) can result in reduced adduction of the knee. This should be explored further in relation to the effects of pregnancy [68].

#### 3.3.3. Rising from a Chair

During the motion of rising from a chair, there are specific stages of flexion and extension that occur to enable locomotion. Normally, the largest moment of hip, ankle, and knee extension takes place at the beginning of the ‘extension’ phase [69]. This is true amongst both pregnant and non-pregnant women. In pregnant women, however, the moment of flexion in the ankle, the peak flexion of the thorax, and the peak flexion of the pelvic segment are increased. Meanwhile, the hip flexion in the third trimester is observed to be significantly lower [70,71]. However, there are similar studies that detect no changes to flexion of the lower limbs, or range of motion of the pelvis, head, and thoracolumbar spine during this sit-to-stand process as a result of pregnancy [70]. In terms of timing, while other stages of gait are observed to take longer during pregnancy, the action of rising from a chair in one study was observed to be shorter as a result of pregnancy, although it took longer in trimesters two and three compared to first trimester measurements [53,71]. The length of the pre-extension phase is reduced with the developing pregnancy, whilst the seat-off time is significantly longer in pregnant women compared to non-pregnant controls [53,70]. Lou et al. found no significant differences in the time taking to stand from a chair [71]. As pregnancy progresses, there is an increase in the velocity of dorsiflexion in the ankle as well as a reduction in the velocity of peak hip extension. Compared to non-pregnant women, pregnant women display a reduced velocity of flexion in the hip [70]. It is important to note that differences in results of these studies may be a result of the chair conditions used. Since it is known that the presence of arms on the chair reduces the maximum flexion in the knee, while increasing the chair height reduces both joint loading and motion in the hip, knee, and ankle across all women (pregnant and non-pregnant), it is likely that these inconsistencies between studies are the reason for varying results [69,71]. Although computational models were developed to analyse the human hip for the normal women subjects [72], there were no studies of [70] biomechanical factors in analysing this significant joint during daily activities. 

#### 3.3.4. Trunk Control during Gait (Pelvic and Thoracic Rotations)

Pregnant women display a reduced range of motion in the trunk during gait, which includes movement of the thorax, pelvis and thoracolumbar spine in the anteroposterior direction [61], which usually leads to certain adaptations in their gait pattern during pregnancy [73]. This could be explained by a more extended thorax, which is observed in pregnant women [28]. The increased extension of the thorax was recorded in a study by McCrory et al., who observed the reduced motion of the trunk in the sagittal plane alongside this [28]. Opposing results were found that in the developing pregnancy, there were no changes to the range of motion of the trunk sagittally [70], yet frontally, a reduced motion of the pelvis was observed as the pregnancy develops. Transverse pelvic-thoracic rotations occur normally during the gait pattern and are described as being in-phase at slow velocities, and slowly become out-of-phase as velocity increases [65]. It is known however that in patients with lower back pain, the rotations continue to be in-phase even at high velocities and this is thought to be the cause of the pain. In one unique study, the pelvic and thoracic rotations of pregnant women were shown to be smaller than those of non-pregnant women; although no change was observed in the developing pregnancy [65]. Furthermore, the pelvic rotations in pregnant women with Pregnancy-related Pelvic Girdle Pain (PPP) were significantly greater than those in pregnant women without PPP [74]. This was also true for the thoracic rotations. The maximum speed attained by PPP women was much lower, which suggested they avoided increasing their speed to avoid the out-of-phase rotations that are normally observed. This could be described as a more ‘careful’ style of walking, perhaps to reduce pain. Tanigawa et al. observed that pregnant women who experience Lumbar Pelvic Pain (LPP) show reduced pelvic-thoracic rotations, since the abdomen has become more rigid in this case, but this varies with the level of pain and the location that it presents [75]. 

### 3.4. Anthropometric Changes

Body mass significantly increases with the developing pregnancy, whilst the trunk becomes longer and abdominal girth significantly increases. This increase in body mass is most significant in the third trimester [42]. Increases can also be observed in the breadth of the thorax, girth of the gluteals, girth of the calves, and biceps and tricipital skin folds during pregnancy. Furthermore, there is an increase of fat in the calves observed as well as a significant reduction in calf muscle. However, changes of this likeness are not observed in the thighs. In terms of the foot, pregnant women display a significantly reduced arch between the 1st and 3rd trimester, as well as a significant increase in the width of the foot [76]. This results in an increased area of contact between the middle of the foot and the floor as well as the lateral heel [7]. An increased pressure in the second metatarsal of the foot was also observed in trimester three compared with both earlier pregnancy and postpartum. Findings also show that pregnant women have higher recorded Foot Posture Indices (FPI) in the third trimester. An increase in FPI describes the foot of a pregnant woman in her late pregnancy [76,77].

It is known that water retention is increased in pregnancy, particularly in the ankles, which is a likely explanation for the increase in foot width and contact with the floor. Also, higher relaxin levels may play a role in relaxing the plantar fascia, the ligament on the sole the foot that supports the arch. A weakened plantar fascia combined with increased weight from pregnancy pushing downwards is a likely explanation for a reduced arch height, and thus an increase in foot contact with the floor. In terms of quality of life, associations have been made between higher reported pain levels in women with flat feet in the general population [78]. 

### 3.5. General Comments on the Quality of the Studies

Most studies identify that participant numbers are a limitation; this is often because of high drop-out rates. However, studies also rarely use participants in their first trimester. This may be because of difficulties surrounding morning sickness in the first trimester [7]. The largest sample size included 110 women, of whom 80 were pregnant and 30 were non-pregnant controls [13]. The smallest sample size was 9 pregnant women [70]. Not only did studies vary in terms of participant numbers, but they greatly varied with regard to gestational weeks. This makes it difficult to compare results between studies, since some separate the pregnancy into three trimesters while others refer to only ‘early’ and ‘late’ pregnancy [61]. However, generally women are analysed in the second and third trimesters (late pregnancy) since little change is observed in studies that include the first trimester. Furthermore, methodologies greatly vary as well as the planes in which each joint is assessed. 

Using the chosen keywords and algorithms, few papers revealed changes in feet or anthropometric changes. Altering the search terms may be useful, since those studies found regarding the feet seem to show significant results. 

Lastly, there is little acknowledgement in studies as to whether the participants are experiencing their first pregnancy or whether they have children already. This may be of importance since it might be likely that increased stressors at home play a role in pain levels, or there may be existing biomechanical changes resulting from a previous pregnancy that create variation between women. 

## 4. Conclusions

There are obvious impacts to the biomechanics of a woman as a result of pregnancy, although most parameters’ results are often conflicting. Despite many non-significant findings, there is evidence to suggest increased angles of lordosis and kyphosis in the spine, as well as increased reports of LBP, although whether or not there is an association needs to be studied further. Reduced trunk motion, static and dynamic stability, gait velocity, hip extension/flexion, foot arch height and increased step width, risk of falls and double support time are commonly reported. It is clear that in cases where significance is found, it is most commonly in the third trimester. Conflicting results are explained in many cases by reasoning that each woman shows individual pregnant morphologies that have varying effects on the biomechanics of the body. Furthermore, each woman adopts unique methods to minimise risk. Therefore, it might be important to assess the individual changes in a pregnant woman, including ankle stiffness and thigh fat area, particularly in her late pregnancy, to understand her own individual risks. Also, studies could investigate whether other individual differences play a role in the effects of pregnancy. These may include number of previous pregnancies, the way the foetus is sat within the uterus, or perhaps even foot size, since we know that contact of feet with the floor is changed by pregnancy. Further studies could also explore frontal spinal curvature, as well as any associations between quality of life and a reduced trunk range of motion and altered joint kinematics.

## Figures and Tables

**Table 1:** Exclusion criteria for the screening stage.

Cardiovascular: 29	Workplace: 4	Kidneys/Renal Function: 24	Cervical Changes: 29	Road Safety: 9
Blood pressure: 24	Pre-eclampsia: 2	Pregnancy behaviours: 11	Treatments: 13	Exercise effects: 14
Animal models: 37	Labor: 6	Stability of proteins: 24	Bipolar disorder: 1	Genetic stability: 4
Social stability: 18	Skin biomechanics: 5	Effects on fetus: 12	No paper found: 7	Diet: 8
Ground reaction forces: 7	Corneal related: 14	Postural orthostatic tachycardia syndrome: 10	Diabetes: 3	Tumour stability: 1
Reliability of pain classification system: 1	Erythrocyte membrane stability: 1	Bacterial stability: 1	Respiratory system: 5	Edema: 1
Seizures: 1	Mental stability: 6	Brain injuries: 1	Hormone stability: 9	Circadian rhythm: 1
Ankylosing spondylitis: 2	Chronic hypoxia: 1	Injury: 2	Achilles reflex: 1	Pelvic insufficiency: 3
Musculoskeletal disorders: 1	Measurement methods: 11	Pelvic organ prolapse: 1	Scoliosis: 1	Geographic analysis: 2
Osteoporosis: 1	Betamimetic effects: 1	Bone formation: 1	HIV: 1	Treating infertility: 1
Uterus: 2	Bladder function: 3	Hyperemesis gravidarum: 1	Magnetic field: 1	Vestibular system: 1
Neuralgia: 1	Postpartum pain: 3	Bone mineral density: 1	Foot changes: 1	Oculomotor: 1

**Table 2:** Timeline of the reviewed articles, number of participants in each study and the main project objectives (P: pregnant, NP: Non-pregnant).

Reference	Title	Year	Participants	Objectives
[22]	The relationship of low back pain to postural changes during pregnancy	1987	34 P	spinal curvature
[24]	Postural changes associated with pregnancy and their relationship with low-back pain	1990	30 P	spinal curvature
[34]	Posture, performance and discomfort in pregnancy	1992	12 P + 12 NP	sitting posture
[37]	Standing working posture compared in pregnant and non-pregnant conditions	1994	27 P + 10 NP	standing posture
[23,79]	Exercise, posture, and back pain during pregnancy	1995	65 P	spinal curvature
[41]	Effect of posture on hip joint moment during pregnancy, while performing a standing task	1996	16 P	hip joint moment
[21]	An analysis of posture and back pain in the first and third trimesters of pregnancy	1998	12 P	spinal curvature, torso kinematics
[38]	A comparison of sitting posture adaptations of pregnant and non-pregnant females	1999	5 P + 5 NP	sitting posture
[71]	Sit-to-stand at different periods of pregnancy	2001	24 P	kinematics of chair rising
[5]	Static trunk posture in sitting and standing during pregnancy and early post partum	2002	9 P + 12 NP	sitting and standing posture
[40]	Effect of pregnancy on trunk range of motion when sitting and standing	2002	9 P + 12 NP	trunk kinematics
[64]	Gait in pregnancy-related pelvic pain: amplitudes, timing, and coordination of horizontal trunk rotations	2008	24 P	trunk and lower limb kinematics and spatiotemporal gait kinematics
[69]	Biomechanical analysis of chair rising in the pregnant woman	2003	30 P	kinematics of chair rising
[65]	Gait coordination in pregnancy: transverse pelvic and thoracic rotations and their relative phase	2004	12 P + 13 NP	trunk kinematics during gait
[6]	Postural equilibrium during pregnancy: decreased stability with an increased reliance on visual cues	2006	12 P + 12 NP	static posture and COP
[47]	Balance (perceived and actual) and preferred stance width during pregnancy.	2008	15 P + 15 NP	COP and balance
[70]	A longitudinal study of the effect of pregnancy on rising to stand from a chair	2008	9 P + 12 NP	kinematics of chair rising
[43]	Postural sway changes during pregnancy: A descriptive study using stabilometry	2009	20 P	static sway changes and COP
[45]	Characteristics of the control of standing posture during pregnancy	2009	35 P + 8 NP	static sway changes
[44]	Dynamic postural stability during advancing pregnancy	2010	41 P + 40 NP	sway changes and COP
[44]	Spinal curvature and characteristics of postural change in pregnant women	2012	15 P + 10 NP	posture - spinal curvature
[44]	Changes of kinematic gait parameters due to pregnancy	2012	13 P	Spatiotemporal gait parameters
[53]	Biomechanics of rising from a chair and walking in pregnant women	2013	12 P + 10 NP	kinematics of chair rising
[63]	Kinematic analysis of gait in the second and third trimesters of pregnancy	2013	22 P + 12 NP	joint kinematics during gait
[57]	Trunk motion and gait characteristics of pregnant women when walking: report of longitudinal study with a control group	2013	9 P	trunk kinematics during gait
[76]	Anthropometric foot changes during pregnancy: a pilot study	2013	10 P	foot parameters
[13]	Evaluation of postural equilibrium and fall risk during pregnancy	2014	80 P + 30 NP	dynamic posture
[28]	The pregnant “waddle”: An evaluation of torso kinematics in pregnancy	2014	41 P + 40 NP	torso kinematics during gait
[50]	Theoretical and experimental indicators of falls during pregnancy as assessed by postural perturbations	2014	14 P + 40 NP	static stability and COP
[25]	Spinal posture and pelvic position during pregnancy: a prospective rasterographic pilot study	2015	13 P + 20 NP	spinal curvature and pelvic/ trunk tilt
[61]	Differences in trunk control between early and late pregnancy during gait	2015	27 P	control of trunk during gait
[46]	Static postural stability in women during and after pregnancy: A prospective longitudinal study	2015	45 P	static stability and COP
[58]	Temporal and spatial parameters of gait during pregnancy	2015	58 P + 23 NP + 9 PP	Spatiotemporal gait parameters
[4]	Changes in the spinal curvature, degree of pain, balance ability, and gait ability according to pregnancy period in pregnant and nonpregnant women	2015	34 P + 15 NP	spinal curvature, balance, spatiotemporal gait parameters, torso kinematics
[59]	Comparison between overweight due to pregnancy and due to added weight to stimulate body mass distribution in pregnancy	2015	18 P + 18 NP	Spatiotemporal gait parameters and joint kinematics
[56]	Adaptive changes in spatiotemporal gait characteristics in women during pregnancy	2016	28 P	Spatiotemporal gait parameters
[27]	Impact of pregnancy on back pain and body posture in pregnant women	2016	26 P	spinal curvature
[26]	Posture and low back pain during pregnancy - 3D study	2016	65 P	spinal curvature
[62]	Three-dimensional kinematic adaptations of gait throughout pregnancy and post-partum	2016	11 P	lower limb kinematics
[42]	Estimation of inertial parameters of the lower trunk in pregnant Japanese women: A longitudinal comparative study and application to motion analysis	2016	8 P + 7 NP	trunk kinematics
[48]	Effects of lower back pain on postural equilibrium and fall risk during the third trimester of pregnancy	2016	68 P	static stability
[66]	Influence of body composition on gait kinetics throughout pregnancy and postpartum period	2016	11 P	Anthropometric changes and joint kinematics
[9]	Pregnancy-related changes in center of pressure during gait	2017	58 P + 23 NP + 9 PP	dynamic sway changes and COP
[51]	Pregnancy and pelvic girdle pain: analysis of centre of pressure during gait	2017	127 P + 22 NP	dynamic stability and COP
[39]	Effects of pregnancy on lumbar motion patterns and muscle responses	2018	34 P + 34 NP	lumbar motion
[7]	Alterations of pregnant gait during pregnancy and post-partum	2018	16 P	gait kinematics and COP
[29]	Changes in gait and posture as factors of dynamic stability during walking in pregnancy	2018	35 P	trunk kinematics and COP
[49]	Changes in posture control of women that fall during pregnancy	2018	100 P	Static stability
[75]	Gait analysis of pregnant patients with lumbopelvic pain using inertial sensor	2018	52 P	trunk kinematics during gait
[77]	Changes in foot posture during pregnancy and their relation with musculoskeletal pain: A longitudinal cohort study	2018	62 P	foot parameters

## Abbreviations

COP	Centre of pressure
LBP	Lower back pain
WDI	Weight distribution index
COM	Centre of mass
AP	Anteroposterior
ML	Mediolateral
APSI	Anteroposterior stability index
OA	Overall stability index
MLSI	Mediolateral stability index
PGP	Pelvic girdle pain
PPP	Pregnancy-related pelvic girdle pain
QOL	Quality of life
